# Factors Associated With a Patient’s Decision to Select a Cost-effective vs the Most Effective Therapy for Their Own Eye Disease

**DOI:** 10.1001/jamanetworkopen.2020.37880

**Published:** 2021-02-22

**Authors:** Danyal Malik, Xuan Cao, Jaron Castillo Sanchez, Tianshun Gao, Jiang Qian, Silvia Montaner, Akrit Sodhi

**Affiliations:** 1 Wilmer Eye Institute, Johns Hopkins University School of Medicine, Baltimore, Maryland; 2 Department of Oncology and Diagnostic Sciences, School of Dentistry, University of Maryland, Baltimore; 3 Department of Pathology, School of Medicine, University of Maryland Marlene and Stewart Greenebaum Cancer Center, Baltimore

## Abstract

**Importance:**

Ten percent of the Medicare Part B budget is spent on aflibercept, used to treat a myriad of ocular neovascular diseases. A substantial portion of these costs can be attributed to a few hundred ophthalmologists, raising concerns regarding the influence of pharmaceutical companies on the choice of medication by a relatively small group of clinicians. One approach to protect patients’ health care interests is to include them in deliberations on the choice of therapy for their eye disease.

**Objective:**

To examine factors associated with patients’ choice between an effective and less expensive off-label drug or a more effective, but also more expensive, US Food and Drug Administration (FDA)–approved drug.

**Design, Setting, and Participants:**

This retrospective cohort analysis used data from the satellite office of a tertiary referral center from August 2, 2013, to April 9, 2018. Insured patients initiating treatment with anti–vascular endothelial growth factor were included in the analysis. Data were analyzed from March 26, 2018, to June 10, 2020.

**Interventions:**

Patients were asked to choose between bevacizumab (approximately $100 per dose), a chemotherapy that is effective, but not FDA approved, for the treatment of ocular vascular disease, or aflibercept (approximately $2000 per dose), an FDA-approved drug for ocular vascular disease that may be more effective than bevacizumab in some patients. Independent of this choice, patients were separately asked by a study coordinator to participate in an invasive clinical study for which they would not be compensated, there was a small risk for an adverse event, and they would not personally benefit from participating (a surrogate marker for altruism).

**Main Outcomes and Measures:**

Factors associated with patients’ choice of medication, including age, sex, ocular disease, race, and participation in an invasive clinical study.

**Results:**

A total of 189 patients were included in the analysis (106 women [56%]; mean [SEM] age, 74.6 [0.8] years). Despite being told that it may not be as effective as aflibercept, 100 patients (53%) selected bevacizumab for their own eye care. An act of altruism (ie, participation in an invasive clinical study) when the patient was making a choice between the 2 drugs was associated with a patient’s choice of bevacizumab (odds ratio [OR], 7.03; 95% CI, 2.27-21.80; *P* < .001); the OR for selecting bevacizumab for patients who never agreed to participate in the clinical study was 0.45 (95% CI, 0.25-0.83; *P* = .001). Age (OR, 1.00; 95% CI, 0.97-1.03; *P* = .86), race (OR, 0.70; 95% CI, 0.41-1.22; *P* = .21), sex (OR, 0.72; 95% CI, 0.39-1.35; *P* = .31), presence of diabetes (OR, 1.52; 95% CI, 0.59-3.93; *P* = .39), and type of eye disease (OR, 0.56; 95% CI, 0.30-1.04; *P* = .07) were not associated with choice of therapy.

**Conclusions and Relevance:**

These findings suggest that clinicians must consider the ethical implications of the influence of altruism when patients participate in the decision between cost-effective vs the most effective medicines for their own health care.

## Key Points

**Question:**

What factors are associated with a patient’s choice between cost-effective and the most effective medicines for their own health care?

**Findings:**

In this cohort study of 189 patients with eye diseases, 100 selected a potentially less effective but also less costly drug for the treatment of their eye disease. A surrogate marker for altruism was the strongest factor associated with this decision.

**Meaning:**

These findings suggest that clinicians should consider the ethical implications of involving patients in selecting their own therapy if altruism is a key driver for this decision.

## Teaser

This cohort study examines factors associated with patients’ choice between an effective and less expensive off-label drug or a more effective, but also more expensive, US Food and Drug Administration–approved drug for treatment of eye disease.

## Introduction

The introduction of therapies targeting vascular endothelial growth factor (VEGF) has had a remarkable effect on the treatment of ocular neovascular disease.^1^ Bevacizumab (Avastin) was the first of the modern anti-VEGF therapies to be available for the treatment of ocular neovascular disease.^2^ Although it was initially approved by the US Food and Drug Administration (FDA) as a cancer chemotherapy in February 2004,^3^ ophthalmologists began using intravitreal injections with bevacizumab as an off-label treatment for neovascular age-related macular degeneration (nvAMD) soon after it became available in the absence of an effective alternative.^4^ Bevacizumab needs to be compounded by a pharmacy for use in the eye, but because the volume used in the eye (50 μL) is a small fraction of the volume in the nearly $3000 bottle used for chemotherapy (16 mL), the cost per injection for bevacizumab is approximately $100 per treatment. Consequently, after its introduction, bevacizumab quickly became the most common treatment for nvAMD.

In June 2006, a closely related drug, ranibizumab (Lucentis), was approved by the FDA specifically for use in the eye and was priced at approximately $3000 per dose. In a clinical trial funded by the National Institutes of Health comparing these 2 therapies for the treatment of nvAMD,^5^ there appeared to be no significant difference in visual acuity outcomes between monthly treatment with bevacizumab and ranibizumab, providing a rationale for clinicians to continue to use the less expensive drug, bevacizumab. A third therapy, aflibercept (Eylea), was subsequently approved by the FDA for use in the eye for approximately $2000 per dose. These 3 anti-VEGF therapies are currently used to treat a myriad of ocular diseases, most notably nvAMD, diabetic eye disease, and retinal vein occlusion (RVO).

Although no agent has a clear clinical advantage over the others in effectiveness or safety,^6^ head-to-head comparisons among these 3 treatments have demonstrated that aflibercept may have a slight benefit over both ranibizumab and bevacizumab in some settings^7^ and is therefore often the first choice for some retina specialists for the treatment of these vision-threatening diseases. Bevacizumab, at a fraction of the cost (approximately 5%), remains the most cost-effective option. Consequently, aflibercept and bevacizumab are the 2 most commonly used therapies for ocular vascular disease.^8^

The role of patients in deciding between the most effective vs the most cost-effective therapy is unclear. In this study, patients undergoing treatment for newly diagnosed vision-threatening eye disease were provided information on the cost and efficacy of 2 anti-VEGF therapies: bevacizumab and aflibercept. They were also told whether their insurance would cover these costs (in part, or in total) and any potential out-of-pocket expenses for each medication. The patients were then asked to choose between these 2 drugs for the treatment of their own eye disease. We then examined which factors were associated with patients’ decision to choose the less expensive bevacizumab over the more effective aflibercept for their own health care. We hypothesized that specific factors (including age, race, sex, diabetes status, ocular disease, and clinical study participation, would be associated with a patient’s decision to choose the less expensive bevacizumab over the more effective aflibercept for their own health care.

## Methods

### Study Design

This retrospective, observational cohort study was conducted at the Wilmer Eye Institute, Johns Hopkins Hospital, Baltimore, Maryland. The protocol was approved by the institutional review board of the Johns Hopkins Medical Institutions and was conducted in accordance with the Declaration of Helsinki.^9^ Patients provided written informed consent to participate. Initial screening identified patients with an ocular vascular disease requiring initiation of anti-VEGF therapy who were seen by a single retinal specialist (A.S.) at a suburban satellite office of the Wilmer Eye Institute from August 2, 2013, to April 9, 2018. The report was prepared in accordance with the Strengthening the Reporting of Observational Studies in Epidemiology (STROBE) reporting guidelines.

### Patients

Patients were informed by the treating physician that they had a choice between 2 similar anti-VEGF medications: (1) bevacizumab, which is not approved by the FDA for use in the eye but is compounded in the hospital pharmacy from an FDA-approved cancer therapy, and which costs approximately $100 per treatment (based on the Medicare allowable charge); or (2) aflibercept, which is approved by the FDA for use in the eye, costs approximately $2000 per treatment (based on the Medicare allowable charge), and has been reported to be more effective than bevacizumab for the treatment of ocular neovascular disease in some settings. The patients were also informed of their share of the cost for each medication based on their insurance coverage.

Separately, all patients were asked by a study coordinator—in the absence of the treating physician—whether they were willing to participate in an invasive clinical study in which they provide an aqueous sample immediately after their injection and (1) they would not receive any compensation for participation in the clinical study; (2) there was a small but real risk for an adverse event; and (3) they would not directly benefit from their participation (ie, participants were told that all samples would be anonymized and that results from studies using their sample could not be traced back to them and would not influence their current or future care). The patients were informed that the purpose of this invasive clinical study was to identify novel biomarkers or therapeutic targets that may, in the future, benefit other patients with ocular neovascular disease.

### Statistical Analysis

Data were analyzed from March 26, 2018, to June 10, 2020. Categorical variables were presented as percentages and compared using the 2-sided χ^2^ test with significance set at *P* < .05. Data for continuous variables were recorded as mean (SEM). Assuming nonparametric data, unpaired, 2-tailed, Mann-Whitney test analysis with significance set at *P* < .05 was used to compare mean data points in this study. Analyses were performed using Prism, version 8 (GraphPad Software).

For the logistic regression of multiple variables, we used a generalized linear model to determine the association between the choice of the drug (aflibercept or bevacizumab) and the 6 listed features: age, race (White, Black, or Asian), sex (female or male), diabetes status (present or absent), ocular disease (nvAMD, RVO, or diabetic eye disease), participation in a clinical study (never, once, or more than once), and participation in the clinical study at their first treatment visit. The choice was set as the binary outcome Y (∈ [0,1]) and the 7 features as the explanatory variables X1, X2, X3, X4, X5, X6, and X7 respectively. The generalized linear model was defined as the linear logistic regression model:

log_10_ (p/1 − p) = β_0_ + β_1_ X1 + β_2_ X2 + β_3_ X3 + β_4_ X4 + β_5_ X5 + β_6_ X6 + β_7_ + X7

where β represents the coefficient for each variable. This method was implemented by the R glm() function in the stats package (R Foundation for Statistical Computing).

## Results

### Patient Demographic and Baseline Characteristics

A review of the medical records of patients seen at a suburban satellite office of a tertiary academic center by a single retina specialist from 2013 to 2018 identified 263 patients with a mean (SEM) age of 74.2 (0.8) who presented with a new diagnosis of an ocular vascular disease requiring initiation of treatment with anti-VEGF therapy (eTable 1 in the Supplement). Patients were informed by their treating physician that they could choose between 2 closely related medications: bevacizumab or aflibercept. The cost and efficacy of each was carefully explained to the patient, as described above. All patients included in this study had insurance and were informed whether they were likely to incur a copayment for the medication (based on their insurance coverage). Importantly, the treating physician did not persuade patients to select one medication over the other, allowing the patients to use their own judgment (and value system) to choose between the 2 drugs.

One hundred eighty-nine patients were qualified for inclusion in this study (eTable 2 in the Supplement). The mean (SEM) age was 74.6 (0.8) years, with 106 women (56%) and 83 men (44%). One hundred fifty-four patients (81%) were White, 18 (10%) were Black, and 17 (9%) were Asian. Most patients (113 [60%]) were being treated for nvAMD, with the remaining patients treated for RVO (40 [21%]) or diabetic eye disease (36 [19%]). These numbers are reflective of the racial diversity of patients in the US treated with anti-VEGF therapy for these 3 diseases.^10^

### Age, Sex, Race, and Diagnosis and a Patient’s Decision to Choose Bevacizumab Over Aflibercept

An approximately equal number of patients selected aflibercept (89 of 189 [47%]) and bevacizumab (100 of 189 [53%]) (*P* = .40) (Table 1). The mean (SEM) age at initial visit (74.8 [1.2] vs 74.5 [1.2] years) was not significantly different between the 2 groups (*P* = .89). Patients 65 years or older (ie, Medicare-eligible patients with full coverage for both medications) were also equally likely to select aflibercept or bevacizumab (76 of 157 [48%] vs 81 of 157 [52%]; *P* = .57). Although women were equally likely to select aflibercept or bevacizumab (55 of 106 [52%] vs 51 of 106 [48%]; *P* = .57), men were less likely to select aflibercept than bevacizumab (34 of 83 [41%] vs 49 of 83 [59%]; *P* = .01). More Black patients selected bevacizumab (13 of 18 [72%]) compared with aflibercept (5 of 18 [28%]; *P* < .001). A diagnosis of a retinal vein occlusion was the only ocular disease that influenced the selection of bevacizumab rather than aflibercept (28 of 40 [70%] vs 12 of 40 [30%]; *P* < .001).

**Table 1.** Presenting Characteristics of All Patients Who Qualified for Inclusion in This Study

Characteristic	Patient selection of therapy^a^		*P* value^b^
	Aflibercept	Bevacizumab	
All	89 (47)	100 (53)	.40
Age, mean (SEM), y	74.8 (1.2)	74.5 (1.2)	.89
Medicare-eligible patients (65 y and older)	76 (48)	81 (52)	.57
Female	55 (52)	51 (48)	.57
Male	34 (41)	49 (59)	.01
Race			
Black	5 (28)	13 (72)	<.001
White	75 (49)	79 (51)	.78
Asian	9 (53)	8 (47)	.40
Disease			
RVO	12 (30)	28 (70)	<.001
Diabetic eye disease	18 (50)	18 (50)	>.99
nvAMD	59 (52)	54 (48)	.57

Abbreviations: nvAMD, neovascular age-related macular degeneration; RVO, retinal vein occlusion.

^a^ Unless otherwise indicated, data are expressed as number (percentage) of row totals.

^b^ Statistical analysis was performed using χ^2^ and Mann-Whitney tests. *P* < .05 indicated significance.

### Participation in a Clinical Trial as a Surrogate Marker for Altruism

Altruism has been defined as any intentional and voluntary action that is designed to increase another person’s (or persons’) welfare without the expectation of reciprocity or compensation for that action and is performed despite a cost to oneself.^11,12,13,14^ We therefore examined altruism as a contributing factor in a patient’s choice of a less effective but more cost-effective medicine for their own health care. To this end, we used an indirect measure, participation in an invasive clinical study, to serve as a surrogate marker for altruism. At each of their clinic visits, all patients included in our study were asked by a study coordinator—without the treating physician present—if they would agree to participate in an invasive clinical study. The study coordinator informed the patients that if they participated in the study (1) they would not be compensated; (2) there was a small risk for an adverse event (ie, a hyphema for all patients and traumatic cataract for patients with phakia) and a possible (theoretical) increased risk for infection when a needle was used to withdraw aqueous fluid from the front of their eye; and (3) they would not personally benefit from the results of the study. In the absence of compensation or access to care or a novel treatment, participation in invasive clinical studies is motivated by altruism, the belief that the well-being of others is equally important as (if not more than) the well-being of the individual. We therefore used agreement to participate in this invasive clinical study as a surrogate marker for altruism.

One hundred twenty-five patients (66%) approached by the study coordinator ultimately agreed to participate in the invasive clinical study (Table 2). Patients who agreed to participate were slightly older compared with those who chose not to participate (mean [SEM] age, 76.2 [0.9] vs 71.5 [1.6] years; *P* = .03). Accordingly, Medicare-eligible patients (65 years or older) were more likely to participate in the invasive clinical trial than to decline participation (109 of 157 [69%] vs 48 of 157 [31%]; *P* < .001). Women were more likely to participate than to decline participation (69 of 106 [65%] vs 37 of 106 [35%]; *P* < .001). Black (11 of 18 [61%] vs 7 of 18 [39%]; *P* = .002) and White (107 of 154 [69%] vs 47 of 154 [31%]; *P* < .001) patients were also more likely to participate than to decline to participate, whereas Asian patients were more likely to decline to participate (10 of 17 [59%] vs 7 of 17 [41%]; *P* = .01) (Table 2). Patients with RVO (30 of 40 [75%] vs 10 of 40 [25%]; *P* < .001) or nvAMD (79 of 113 [70%] vs 34 of 113 [30%]; *P* < .001) were also more likely to participate than decline.

**Table 2.** Characteristics of All Patients Who Agreed to Participate in a Clinical Study

Characteristic	Participation group^a^		*P* value^b^
	Participants	Nonparticipants	
All	125 (66)	64 (34)	<.001
Age, mean (SEM), y	76.2 (0.9)	71.5 (1.6)	.03
Medicare-eligible patients (65 y or older)	109 (69)	48 (31)	<.001
Female	69 (65)	37 (35)	<.001
Male	56 (67)	27 (33)	<.001
Race			
Black	11 (61)	7 (39)	.002
White	107 (69)	47 (31)	<.001
Asian	7 (41)	10 (59)	.01
Disease			
RVO	30 (75)	10 (25)	<.001
Diabetic eye disease	16 (44)	20 (56)	.09
nvAMD	79 (70)	34 (30)	<.001

Abbreviations: nvAMD, neovascular age-related macular degeneration; RVO, retinal vein occlusion.

^a^ Unless otherwise indicated, data are expressed as number (percentage) of row totals.

^b^ Statistical analysis was performed using χ^2^ and Mann-Whitney tests. *P* < .05 indicated significance.

### Altruistic Behavior and the Choice Between Aflibercept and Bevacizumab

When we examined the subset of patients who agreed to participate in the invasive clinical study (n = 125), a roughly equal number selected aflibercept and bevacizumab (57 [46%] vs 68 [54%], respectively; *P* = .26) (Table 3). Black (4 of 11 [36%] vs 7 of 11 [64%]; *P* < .001) and Asian (2 of 7 [29%] vs 5 of 7 [71%]; *P* < .001) patients who elected to participate in the study were more likely to select bevacizumab over aflibercept by a margin of roughly 2:1 (Table 3), whereas White patients were equally likely to choose aflibercept and bevacizumab (51 of 107 [48%] vs 56 of 107 [52%], respectively; *P* = .57). Patients with RVO who elected to participate in the study were more likely to select bevacizumab than aflibercept (8 of 30 [27%] vs 22 of 30 [73%]; *P* < .001), but patients with diabetic eye disease (8 of 16 [50%] vs 8 of 16 [50%], respectively; *P* > .99) or nvAMD (41 of 79 [52%] vs 38 of 79 [48%], respectively; *P* = .57) were equally likely to select bevacizumab or aflibercept. Collectively, these results suggested that for some patients, altruism may contribute to the decision to select a less effective but more cost-effective medicine for their own health care. However, altruism did not appear to be a primary driver for this decision for most patients.

**Table 3.** Characteristics of Patients Who Agreed to Participate in a Clinical Study and Who Selected Aflibercept or Bevacizumab for the Treatment of Their Ocular Vascular Disease

Characteristic	Patient selection of therapy^a^		*P* value^b^
	Aflibercept	Bevacizumab	
All	57 (46)	68 (54)	.26
Age, mean (SEM), y	76.3 (1.3)	76.1 (1.3)	.995
Medicare-eligible patients (65 y and older)	51 (47)	58 (53)	.40
Female	35 (51)	34 (49)	.78
Male	22 (39)	34 (61)	.002
Race			
Black	4 (36)	7 (64)	<.001
White	51 (48)	56 (52)	.57
Asian	2 (29)	5 (71)	<.001
Disease			
RVO	8 (27)	22 (73)	<.001
Diabetic eye disease	8 (50)	8 (50)	>.99
nvAMD	41 (52)	38 (48)	.57

Abbreviations: nvAMD, neovascular age-related macular degeneration; RVO, retinal vein occlusion.

^a^ Unless otherwise indicated, data are expressed as number (percentage) of row totals.

^b^ Statistical analysis was performed using χ^2^ and Mann-Whitney tests. *P* < .05 indicated significance.

### Altruistic Behavior at the Time of Drug Selection and the Choice Between Bevacizumab and Aflibercept

Although initially an altruistic act was thought to be intuitive, reflexive, and even automatic,^15^ recent evidence suggests that it is instead a thoughtful choice with pros, cons, and even occasional mistakes, just like any other choice.^16^ Because a person’s altruistic vs egoistic (ie, selfish) drives are conditional and often situational, we examined whether an act of altruism that coincided with the time when they chose between aflibercept and bevacizumab was better associated with a patient’s decision to select a less effective but more cost-effective medicine for their own health care. Interestingly, although similar numbers of patients who ultimately agreed to participate in the invasive clinical study selected aflibercept and bevacizumab (57 of 89 [64%] vs 68 of 100 [68%], respectively; *P* = .26), significantly more patients who agreed to participate in the invasive clinical study at their initial treatment visit (when they were choosing between the 2 medications) selected bevacizumab than aflibercept (61 of 99 [62%] vs 38 of 99 [38%], respectively; *P* = .001) (Table 4).

**Table 4.** Patients Who Chose Aflibercept or Bevacizumab and Who Agreed to Participate in a Clinical Trial (ie, a Surrogate Marker for Altruism)

Characteristic	Patient selection of therapy, no. (%)		*P* value^a^
	Aflibercept	Bevacizumab	
All (N = 189)	89 (47)	100 (53)	NA
Patients who participated during their initial treatment	38 (38)	61 (62)	.001
Patients who participated at least once during any treatment	57 (46)	68 (54)	.26

Abbreviation: NA, not applicable.

^a^ Statistical analysis was performed using χ^2^ and Mann-Whitney tests. *P* < .05 indicated significance.

### Multiple Regression Analysis for Key Factors Associated With Selection of Bevacizumab Over Aflibercept

Collectively, these results suggest that altruism, specifically an altruistic act at the time when the patient was deciding which drug to choose for their own health care, influenced patients’ decision to select bevacizumab over aflibercept. To determine the importance of altruism compared with other factors, we used multiple regression analyses to correlate the patient’s choice of medication with age, sex, ocular disease, race, diabetes status, and their agreement to participate in the invasive clinical study at their first visit or at any visit (Table 5). We observed that the odds ratio (OR) for selecting bevacizumab for patients who agreed to participate in an invasive clinical study on their first treatment visit, when they made their choice of medication, was 7.03 (95% CI, 2.27-21.80; *P* < .001). Conversely, the OR for selecting bevacizumab for patients who never agreed to participate in the clinical study was 0.45 (95% CI, 0.25-0.83; *P* = .01). Associations for age (OR, 1.00; 95% CI, 0.97-1.03; *P* = .86), race (OR, 0.70; 95% CI, 0.41-1.22; *P* = .21), sex (OR, 0.72; 95% CI, 0.39-1.35; *P* = .31), presence of diabetes (OR, 1.52; 95% CI, 0.59-3.93; *P* = .39), and type of eye disease (OR, 0.56; 95% CI, 0.30-1.04; *P* = .07) were not significant. Collectively, these results identify altruism as the strongest factor associated with a patient’s decision to select a less effective but more cost-effective medicine for their own health care.

**Table 5.** Logistic Regression of Multiple Variables Demonstrating Odds of a Patient Selecting Bevacizumab Over Aflibercept

Variable	Coefficient (estimate)	Adjusted OR (95% CI)	*P* value (Wald test)^a^	*P* value (LR test)^a^
Age	–0.0027	1.00 (0.97-1.03)	.86	.86
Race	–0.3502	0.7 (0.41-1.22)	.21	.21
Sex	–0.3249	0.72 (0.39-1.35)	.31	.31
Diabetes status	0.4176	1.52 (0.59-3.93)	.39	.39
Disease	–0.5826	0.56 (0.30-1.04)	.07	.06
Patients who did not volunteer to participate during the study	–0.7887	0.45 (0.25-0.83)	.01	.005
Participation at first treatment visit	1.9503	7.03 (2.27-21.8)	<.001	<.001

Abbreviations: LR, logistic regression; OR, odds ratio.

^a^ *P* < .05 indicates significance.

## Discussion

It is essential that patients participate in their own health care decisions.^17^ Inclusion of patients in the informed decision on how to distribute health care resources is in line with evidence-based patient choice^18^ and may further provide balance to the deliberations on which medication a patient should receive for the treatment of their own eye disease. This is particularly true when there is reasonable disagreement among physicians—and patients—as to which treatment is most appropriate for their disease.^19^ However, this decision is more complex when the patient is asked to choose between 2 similar medications: one that appears to be less effective but also costs less, and another that is more expensive but may also be more effective. This choice is exemplified by the decision between bevacizumab and aflibercept.

Herein, patients initiating anti-VEGF therapy were asked to decide which medication, aflibercept or bevacizumab, they should receive for their own eye care. To facilitate an informed decision, patients were told that bevacizumab is a chemotherapy that is effective, but not approved by the FDA, for the treatment of ocular vascular disease and costs approximately $100 per dose, and that aflibercept is an FDA-approved drug for ocular vascular disease that costs approximately $2000 per dose but in some settings may be more effective than bevacizumab. Careful attention was paid during the presentation of these 2 options not to bias patients toward one choice over the other. Given this information, 100 of 189 patients (53%) selected bevacizumab over aflibercept for their own eye care.

Independent of this choice, patients were asked by a study coordinator to participate in an invasive clinical study. In the absence of compensation or access to care or a novel treatment, participation in invasive clinical studies is motivated by altruism, the belief that the well-being of others is equally important as (if not more important than) the well-being of the individual. Interestingly, an equal and remarkably high fraction of patients (125 of 189 [66%]) ultimately agreed to participate in the invasive clinical study, suggesting that the patients who chose bevacizumab were not necessarily inherently more altruistic than those who chose aflibercept. However, at the time of their decision between the 2 medications, there was an association between those patients who agreed to participate in the invasive clinical study and those who chose bevacizumab (OR, 7.03; *P* < .001). This finding supports prior studies demonstrating that altruism is situational.^20^ Conversely, patients who elected not to participate in the clinical study when approached at each of their visits were more likely to select aflibercept (OR, 0.45; *P* = .01).

We observed that Black participants in our study strongly favored selecting bevacizumab over aflibercept (72% vs 28%; *P* < .001). A number of factors (eg, socioeconomic considerations, insurance coverage, and perceptions of wastefulness) may have been associated with this favorability toward bevacizumab. However, we cannot rule out the possibility that Black patients favored bevacizumab owing to altruism.

### Limitations

This study has some limitations. One limitation of using a patient’s decision to volunteer for a clinical study as a surrogate marker for altruism is that it assumes that all altruistic patients are equally likely to volunteer for a clinical trial. However, the historical treatment of Black individuals in medical research resulted in a general skepticism about participating in clinical trials. In our study, we may therefore have underestimated the influence of altruism in motivating Black patients to select bevacizumab if they chose not to participate in the clinical study owing to this skepticism.

Additional limitations of our study include that it is a retrospective study with a limited number of patients. We also did not directly survey patients to assess their motivation for selecting bevacizumab over aflibercept but instead used a surrogate marker for altruism (ie, participation in an invasive clinical study). However, patients were told by their physician that both medications were good choices and that half of the physician’s patients choose one medication and half choose the other; there is no wrong choice. The physician was also not present and did not participate in the discussions about the invasive clinical study; this duty was performed independently by the study coordinator. In this context, an independent altruistic act serves as a surrogate marker for altruism. Nevertheless, we acknowledge that despite our efforts, there is no ideal measurement for altruism, and other factors (fear of high out-of-pocket expenses, innate desire to please their treating physician, or a sense of shame for choosing the more costly medication) could also have influenced this decision.

## Conclusions

The results of this cohort study have broad implications about patient participation in the decision between the most effective or the most cost-effective medicine for their own health care. Is it fair for a self-selected group of patients to shoulder the burden of mitigating health care costs (or improving cost-efficiency) while others can choose not to do so? It is reasonable to argue that an appeal to altruism is coercive and puts society’s interests above those of the individual patient.^21^ Insurance companies may be motivated by profits, and clinicians can be influenced by pharmaceutical companies.^22^ Our observations suggest that asking patients to contribute to these deliberations, while enabling evidence-based patient choice,^18^ may also result in an unequal (and, arguably, unfair) allocation of medical resources among patients with similar diseases. This possibility is highlighted by the observation that patients with RVO—whose visual outcome may benefit more from the longer-acting anti-VEGF therapy, aflibercept—were more likely to select bevacizumab over aflibercept. Collectively, our results demonstrate that clinicians need to consider the consequences of asking patients to participate in the decision of how to distribute scarce health care resources when it comes to their own health care.

## Notes

Supplement. **eTable 1.** Baseline Characteristics of All Patients Who Presented Between 2013 and 2018 With a New Diagnosis of Ocular Vascular Disease Requiring Initiation of Anti-VEGF Therapy **eTable 2.** Presenting Characteristics of All Patients Who Qualified for Inclusion in This Study
