# Supplementary material for: Factors Associated With a Patient’s Decision to Select a Cost-effective vs the Most Effective Therapy for Their Own Eye Disease
Source: JAMA Netw Open. 2021 Feb 22;4(2):e2037880. doi: 10.1001/jamanetworkopen.2020.37880 (PMC7900858; doi:10.1001/jamanetworkopen.2020.37880)
Supplement: Supplement. — eTable 1. Baseline Characteristics of All Patients Who Presented Between 2013 and 2018 With a New Diagnosis of Ocular Vascular Disease Requiring Initiation of Anti-VEGF Therapy eTable 2. Presenting Characteristics of All Patients Who Qualified for Inclusion in This Study [file jamanetwopen-e2037880-s001.pdf]

Supplementary Online Content

Malik D, Cao X, Sanchez JC, et al. Factors associated with a patient's decision to select a cost-effective vs the most effective therapy for their own eye disease. *JAMA Netw Open*. 2021;4(2):e2037880. doi:10.1001/jamanetworkopen.2020.37880

eTable 1. Baseline Characteristics of All Patients Who Presented Between 2013 and 2018 With a New Diagnosis of Ocular Vascular Disease Requiring Initiation of Anti-VEGF Therapy

eTable 2. Presenting Characteristics of All Patients Who Qualified for Inclusion in This Study

This supplementary material has been provided by the authors to give readers additional information about their work.

©2021 Malik D et al. *JAMA Network Open*.

eTable 1. Baseline Characteristics of All Patients Who Presented Between 2013 and 2018 With a New Diagnosis of Ocular Vascular Disease Requiring Initiation of Anti-VEGF

**Characteristic**		
Total Patients Screened		263
Mean Age (years)		74.2 ± 0.8
Females – no. (%)		145 (55)
Race – no. (%)	African American	22 (8)
	Caucasian	215 (82)
	Asian	26 (10)
Disease – no. (%)	RVO	57 (22)
	Diabetic Eye Disease	46 (18)
	nvAMD	160 (60)

Abbreviations: No., number; RVO, Retinal Vein Occlusion (includes branch, hemi-, and central RVOs). nvAMD (neovascular age-related macular degeneration). Values displayed as mean ± standard error of the mean. Statistical analysis was performed using Chi-square and Mann-Whitney

©2021 Malik D et al. *JAMA Network Open*.

eTable 2. Presenting Characteristics of All Patients Who Qualified for Inclusion in This Study

**Characteristic**		
Total Patients Screened		189
Mean Age (years)		74.6 ± 0.8
Females – no. (%)		106 (56)
Race – no. (%)	African American	18 (10)
	Caucasian	154 (81)
	Asian	17 (9)
Disease – no. (%)	RVO	40 (21)
	Diabetic Eye Disease	36 (19)
	nvAMD	113 (60)

Abbreviations: No., number; RVO, Retinal Vein Occlusion (includes branch, hemi-, and central RVOs). nvAMD (neovascular age-related macular degeneration). Values displayed as mean ± standard error of the mean. Statistical analysis was performed using Chi-square and Mann-Whitney test. Data in bold are statistically significant.

©2021 Malik D et al. *JAMA Network Open*.
